# Uhrf1 regulates H3K9me2 modification of mTOR to inhibit the effect of autophagy in myocardial ischemia-reperfusion injury

**DOI:** 10.18632/aging.202722

**Published:** 2021-03-19

**Authors:** Han-Geng Li, Wen-Hua Tian, Cun-Lan Qin, Rong-Rong Ye, Dong-Hua Liu, Hui-Wen Liu

**Affiliations:** 1 Department of Histology and Embryology, College of Basic Medicine, Harbin Medical University, Harbin 150081, China; 2 Emergency Department, The Fourth Hospital of Harbin Medical University, Harbin 150081, China

**Keywords:** Uhrf1, myocardial ischemia-reperfusion injury, mTOR, H3K9me2, autophagy

## Abstract

The regulation of mTOR and the dimethylation of histone H3 on lysine 9 (H3K9me2) H3K9me2 by Uhrf1 and the mechanism of autophagy regulation in myocardial ischemia-reperfusion injury (MIRI) were studied *in vivo* and *in vitro*.

An *in vitro* I/R injury model was established using the primary mouse cardiomyocytes treated with H_2_O_2_. Subsequent analysis by qRT-PCR, western blot, and immunofluorescence indicated that overexpression of Uhrf1 significantly inhibited apoptosis of the H_2_O_2_-treated cardiomyocytes, reduced expression of apoptosis factors caspase-3 and Bax, and increased expression of apoptosis inhibitory factor Bcl-2. Furthermore, Uhrf1 was found to increase cardiomyocyte proliferation and promote the expression of mTOR, while the four expression peaks of H3K9me2 on the mTOR gene were inhibited by overexpression of Uhrf1. The expression of autophagy factors LC3, Beclin-1, and p-mTOR in Uhrf1-overexpressed cardiomyocytes was dramatically increased, and P62 expression was dramatically decreased. When an H3K9me2 inhibitor was added to the Uhrf1-knockdown cardiomyocytes, the expression of mTOR was increased, the expression of LC3, Beclin-1, and p-mTOR was decreased, and P62 expression was significantly increased.

In the present study, Uhrf1 exhibits a protective function in MIRI, reducing the apoptosis of cardiomyocytes while increasing their proliferation and viability.

## INTRODUCTION

Myocardial ischemia-reperfusion injury (MIRI) is a pathological process of progressive aggravation of ischemic myocardium after partial or complete coronary artery occlusion. With the development of basic research and clinical treatments, coronary artery bypass grafting, percutaneous coronary angioplasty, and intravenous thrombolysis have become widely used in clinical practice. However, MIRI limits the success rate of these treatments. A series of damaging changes in cardiac function electrophysiology, myocardial ultrastructure, and energy metabolism, and even severe arrhythmia, caused by ischemia can lead to sudden death. Timely reperfusion is the most effective method of salvation from myocardial ischemia [1]. However, a series of adverse events often caused rapid reperfusion, such as endoplasmic reticulum stress, Ca^2+^ overload, and excessive reactive oxygen species (ROS) production. The adverse events mentioned before are generally specified as ischemia/reperfusion (I/R) injury [2, 3].

Ubiquitin like with PHD and ring finger domains 1 (Uhrf1) is an epigenetic modifier which is highly expressed in many tumor cells. It has been shown involved in cell apoptosis and proliferation, but its role in cardiomyocytes has not been reported.

Accordingly, in this research, I/R models were established to determine the relationship between Uhrf1 and MIRI *in vitro* and *in vivo*. Additionally, we investigated the function of Uhrf1 in myocardial I/R and its relationship to mammalian target of rapamycin (mTOR). Uhrf1 regulates mTOR by adjusting H3K9 methylation (H3K9me2). The alleviative effects of the Uhrf1/mTOR/ autophagy axis on MIRI were also revealed.

## RESULTS

### Changes in Uhrf1 expression in the *in vivo* MIRI mouse model

**Figure 1:**
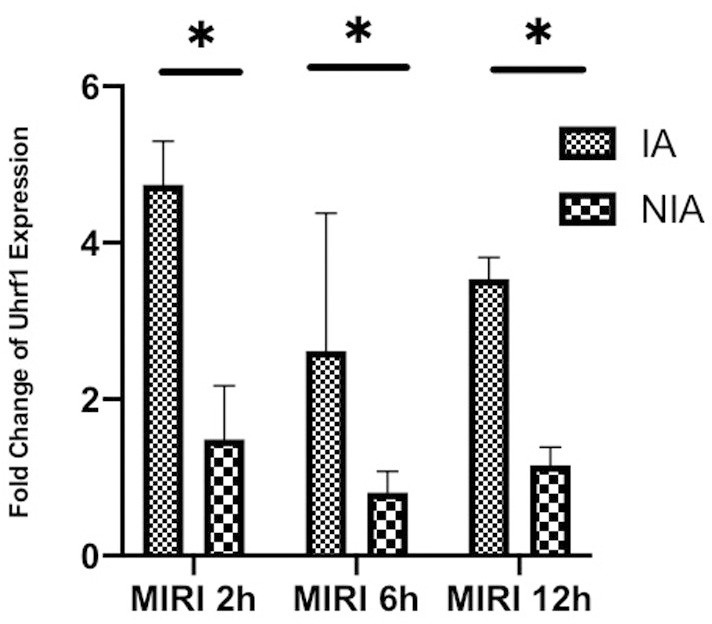
**Detection of the changes of Uhrf1 in myocardial ischemia-reperfusion injury model of mice *in vivo*.** The relative mRNA expressions of Uhrf1 in the infarcted and non-infarcted regions of the mice hearts were detected by qRT-PCR at 2, 6 and 12 h after myocardial ischemia-reperfusion injury. Data shown are mean ± SD. * *P* < 0.05, ** *P* < 0.01, *** *P* < 0.001, **** *P* < 0.0001. N=3 per group.

The mice were divided into three groups, which were subjected to myocardial ischemia for 30 minutes and then reperfusion for 2, 6, or 12 hours. The expression of Uhrf1 in the infarcted areas (IA) and non-infarcted areas (NIA) were detected by qRT-PCR (Figure 1). The Uhrf1 level in the non-infarcted areas was significantly lower than the infarcted areas at 2, 6 and 12 hours after myocardial ischemia-reperfusion *in vivo*.

### Protective effect of Uhrf1 in MIRI

**Figure 2:**
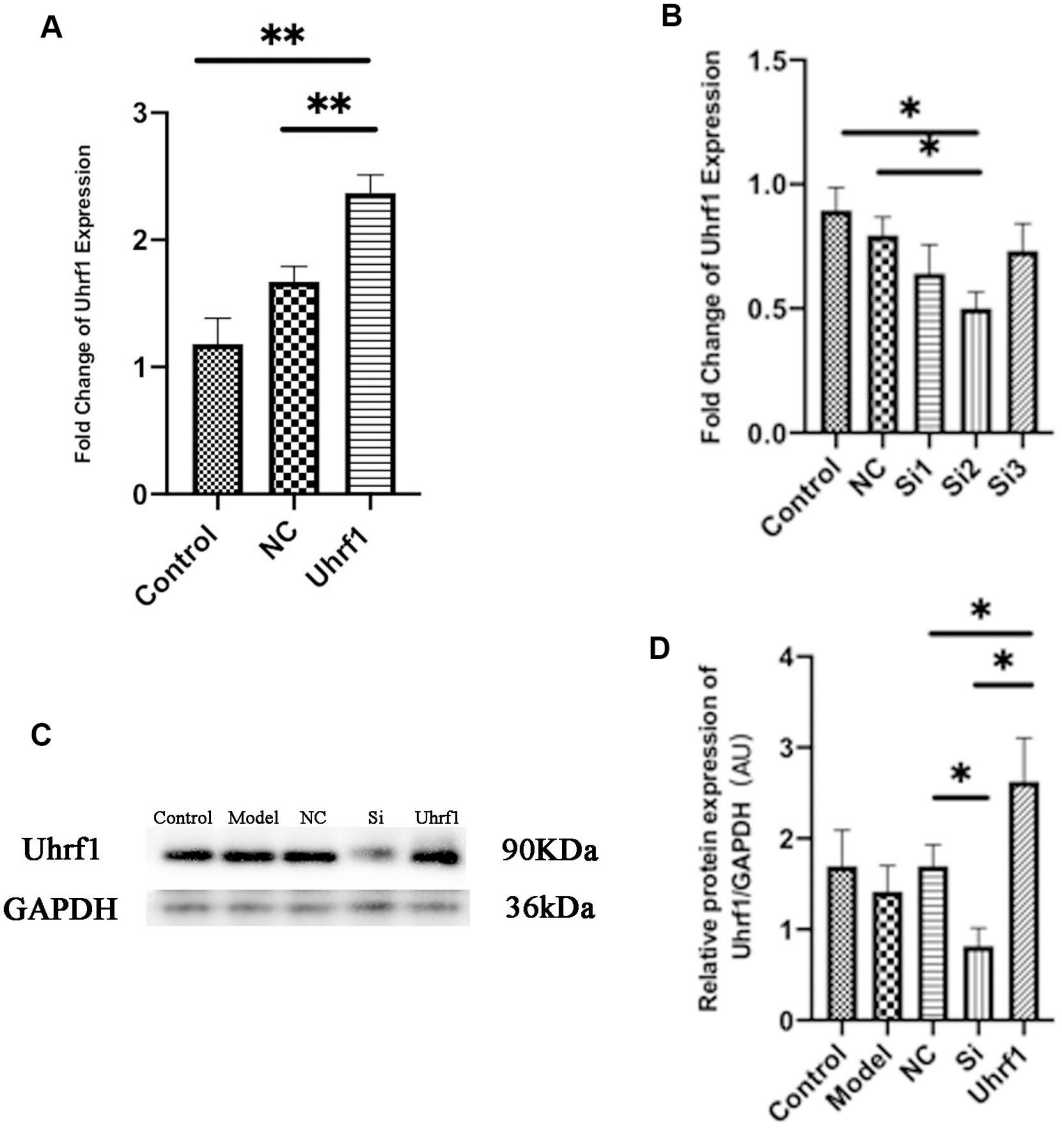
**Detection of transfection efficiency of overexpression and knockdown of Uhrf1 plasmid.** (**A**) The relative mRNA expressions of Uhrf1 in Myocardial ischemia-reperfusion model *in vitro* with Uhrf1 plasmid were determined by qRT-PCR. NC, negative control of plasmid transfection; Uhrf1, Uhrf1 overexpression. (**B**) The relative mRNA expressions of Uhrf1 in Myocardial ischemia-reperfusion model *in vitro* with Uhrf1 siRNA plasmid were determined by qRT-PCR. NC, negative control of RNAi; si1-3, siRNA targeting different mRNA regions. (**C**) Western blot was used to detect the expression level of Uhrf1 protein in each group. GAPDH serves as a loading control. Model, *in vitro* oxidative stress model; NC, negative control of RNAi; si, RNAi knockdown of Uhrf1; Uhrf1, Uhrf1 overexpression. (**D**) Expression of Uhrf1 protein relative to GAPDH data from 3 biological repeats is shown. Data shown are mean ± SD. *P < 0.05, **P < 0.01, ***P < 0.001, ****P < 0.0001. N=3 per group.

The protective effect of Uhrf1 on MIRI was investigated by comparative analysis of overexpression and knockdown of Uhrf1 in an *in vitro* MIRI model. To establish stable cardiomyocytes that exhibit overexpression and knockdown of Uhrf1, we analyzed the expression of Uhrf1 plasmid transfection and screened the knockdown efficiency of different siRNAs on Uhrf1. The sequences for siRNA were in Table 1. The efficacy of overexpression and knockdown of Uhrfl was verified by qRT-PCR (Figure 2A, 2B) and western blot (Figure 2C, 2D). We found the protein level of Uhrf1 increased 1.4 fold in the overexpression cells and siRNA-002 (Si2) significantly knocked down the expression of Uhrf1 in cardiomyocytes. In subsequent *in vitro* experiments, Si2 was used to knock down the Uhrf1 (Si group).

**Table 1:** siRNA sequences used in this study.

**Product ID**	**Name**	**Target sequences**
siG171020083716	si-m-Uhrf1_001	GCAACATCAGGCTCTTGAA
siG160511081008	si-m-Uhrf1_002	CCTTGCAGACCATTCTCAA
siG171020083708	si-m-Uhrf1_003	GATGATTGAGCTCCCTAAA

**Figure 3:**
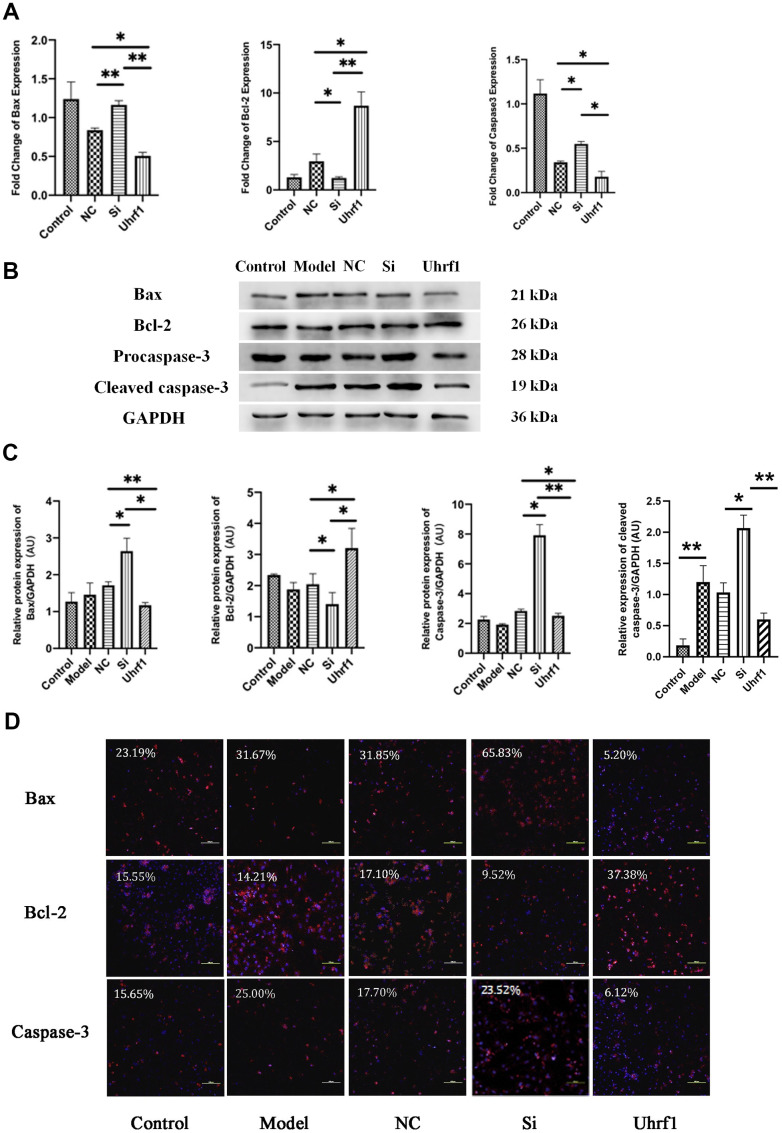
**The effect of Uhrf1 on cardiomyocyte apoptosis during myocardial ischemia-reperfusion injury.** (**A**) The relative mRNA expressions of Bax, Bcl-2 and Caspase-3 in myocardial ischemia-reperfusion model *in vitro* were determined by qRT-PCR. (**B**) Western blot was used to detect the expression level of Bax, Bcl-2 and Caspase-3 protein in each group. GAPDH serves as a loading control. (**C**) Expression of Bax, Bcl-2 and Caspase-3 protein relative to GAPDH data from 3 biological repeats is shown. (**D**) Detection of Bax, Bcl-2 and Caspase-3 protein expression and semi quantitative analysis by immunofluorescence microscopy (x200). Scale bar, 100 μm. Data shown are mean ± SD. *P < 0.05, **P <0.01, ***P <0.001, ****P < 0.0001. N=3 per group. Model, *in vitro* oxidative stress model; NC, negative control of RNAi; si, RNAi knockdown of Uhrf1; Uhrf1, Uhrf1 overexpression.

To confirm the effect of Uhrf1 in cardiomyocyte apoptosis, the mRNA and protein levels of Bcl-2, procaspase-3, cleaved caspase-3 and Bax (Figure 3A) were determined by qRT-PCR, western blot (Figure 3B, 3C) and immunocytochemistry analyses (Figure 3D). Our results show that overexpression of Uhrf1 reduces cardiomyocyte apoptosis after MIRI, decreases the expression of the apoptosis factors Bax, pro- and cleaved caspase-3 at both the mRNA and protein levels, and increases the expression of apoptosis inhibitory factor Bcl-2. Knockdown of Uhrf1in cardiomyocyte showed the opposite trend after MIRI.

**Figure 4:**
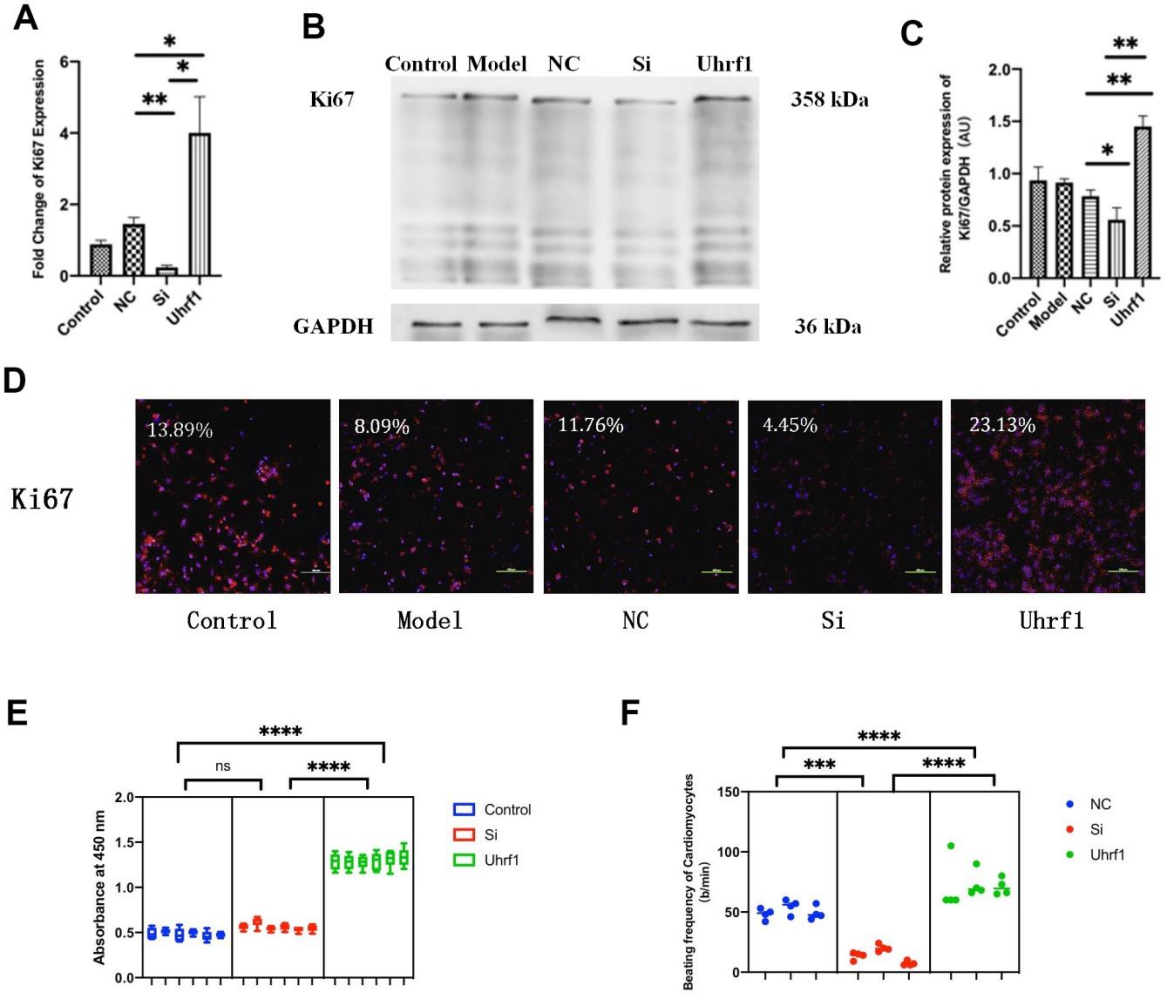
**The effect of Uhrf1 on cell cycle and viability in myocardial ischemia-reperfusion injury.** (**A**) The relative mRNA expressions of Ki67 in Myocardial ischemia-reperfusion model *in vitro* were determined by qRT-PCR. (**B**) Western blot was used to detect the expression level of Ki67 protein in each group. GAPDH serves as a loading control. (**C**) Expression of Ki67 protein relative to GAPDH data from 3 biological repeats is shown. (**D**) Detection of Ki67 protein expression and semi quantitative analysis by immunofluorescence microscopy (x200). Scale bar, 100μm. (**E**) The cardiomyocytes viability in knockdown and overexpression Uhrf1 groups was detected by CCK8. (**F**) The beating times per minute of cardiomyocytes in overexpression and knockdown group. Data shown are mean ± SD. *P <0.05, **P <0.01, ***P <0.001, ****P <0.0001. N=3 per group. Model, *in vitro* oxidative stress model; NC, negative control of RNAi; si, RNAi knockdown of Uhrf1; Uhrf1, Uhrf1 overexpression.

To explore the regulatory effect of Uhrf1 on the cell cycle and the activity of cardiomyocytes, we measured the expression of Ki67 (Figure 4A–4D), the activity of cardiomyocytes (Figure 4E), and the beating frequency of cardiomyocytes in each group (Figure 4F). Our results show that overexpression of Uhrf1 in MIRI increases Ki67 expression at both the mRNA and protein levels. Knockdown of Uhrf1 showed the opposite trend, suggesting that Uhrf1 can increase cardiomyocytes proliferation after MIRI. Using CCK8 to detect cell viability, we found that cardiomyocytes overexpression of Uhrf1 significantly increased the cell viability after MIRI, and there was no significant change in the viability of cardiomyocytes with Uhrf1 knockdown. Moreover, we observed that cardiomyocytes over-expressing Uhrf1 had higher beating frequency than the blank plasmid control after MIRI, while knockdown of Uhrf1 in cardiomyocytes resulted in lower beating frequency than the blank plasmid control.

### Effect of Uhrf1 on the expression of mTOR during MIRI

**Figure 5:**
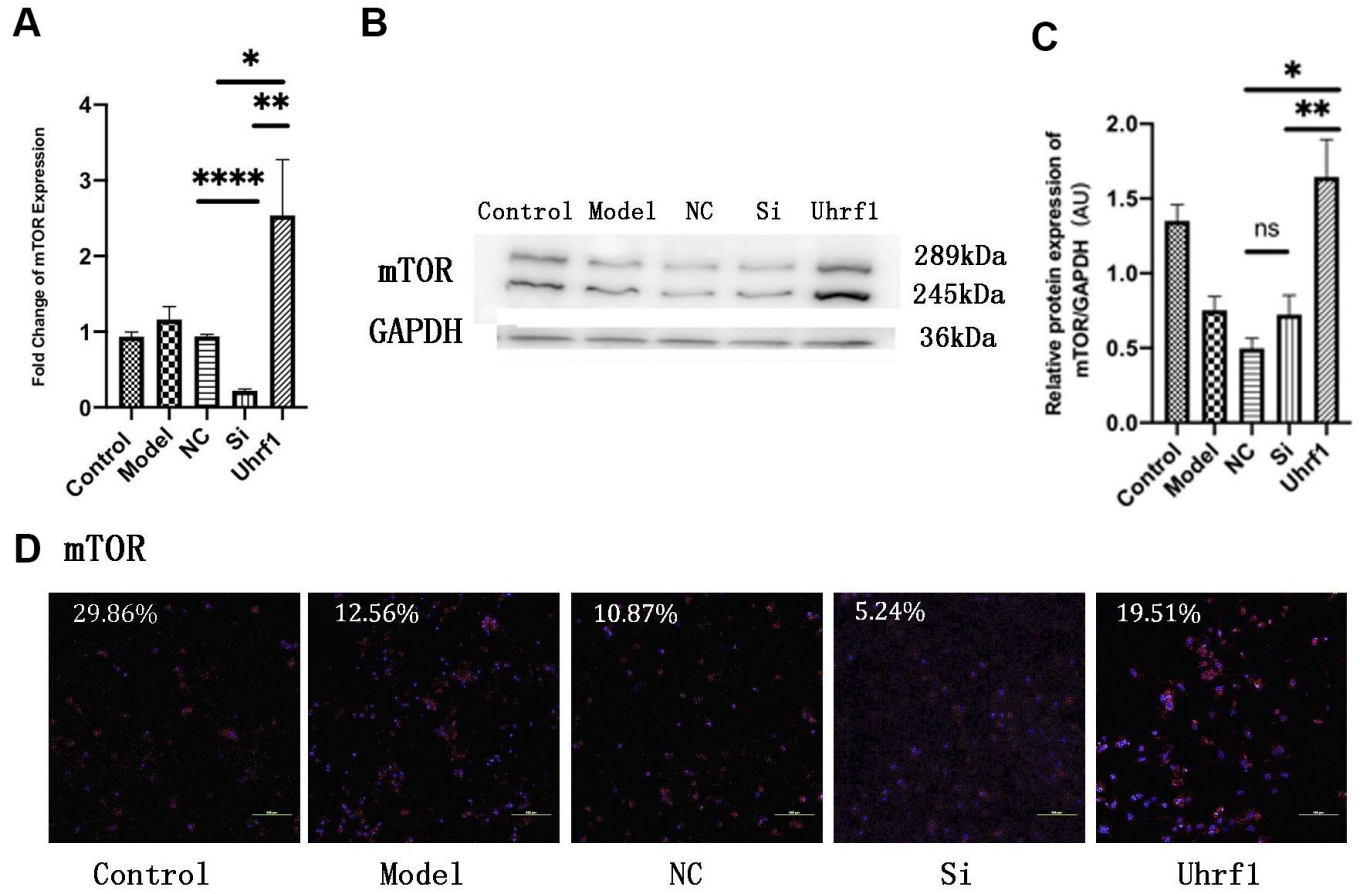
**Uhrf1 promotes the expression of mTOR in MIRI.** (**A**) The relative mRNA expressions of mTOR in Myocardial ischemia-reperfusion model *in vitro* were determined by qRT-PCR. (**B**) Western blot was used to detect the expression level of mTOR protein in each group. GAPDH serves as a loading control. (**C**) Expression of mTOR protein relative to GAPDH data from 3 biological repeats is shown. (**D**) Detection of mTOR protein expression and semi quantitative analysis by immunofluorescence microscopy (x200). Scale bar, 100μm. Data shown are mean ± SD. * *P* < 0.05, ** *P* < 0.01, *** *P* < 0.001, **** *P* < 0.0001. N=3 per group. Model, *in vitro* oxidative stress model; NC, negative control of RNAi; si, RNAi knockdown of Uhrf1; Uhrf1, Uhrf1 overexpression.

To investigate the regulatory effect of Uhrf1 on mTOR in MIRI, we used qRT-PCR to detect changes in the mRNA levels of mTOR after overexpression and knockdown of Uhrf1 (Figure 5A) and confirmed the results by western blot (Figure 5B, 5C) and immunofluorescence (Figure 5D) analysis. We observed that overexpression of Uhrf1 increases mTOR expression at both the mRNA and protein levels. In contrast, knockdown of Uhrf1 only reduced mTOR expression in the mRNA level, and there was no significant change in the protein levels.

### Uhrf1 regulates mTOR expression through H3K9me2

**Figure 6:**
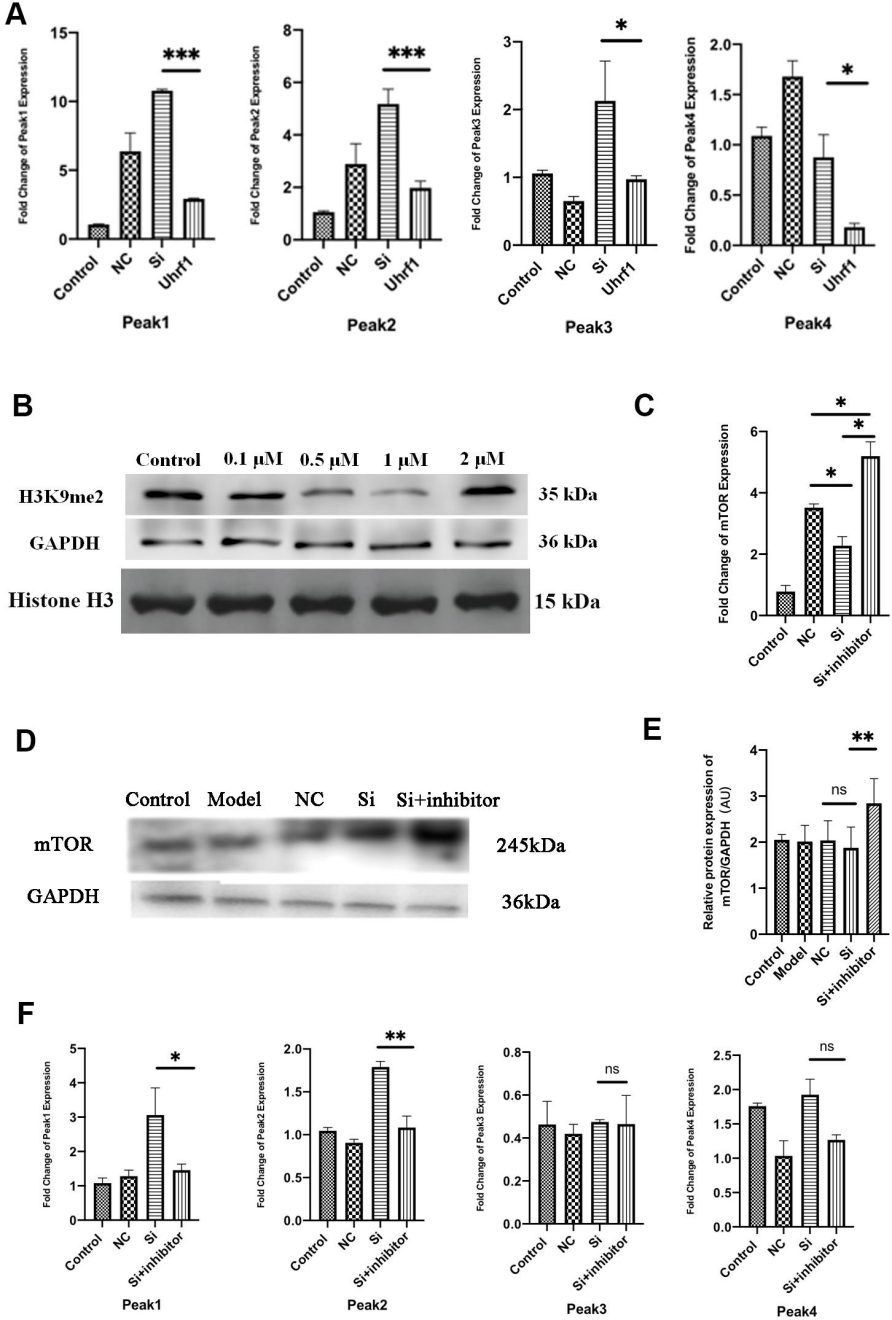
**Uhrf1 regulates mTOR expression through H3K9me2.** (**A**) The relative mRNA expressions of Peak1-4 in Myocardial ischemia-reperfusion model *in vitro* were determined by qRT-PCR. (**B**) Western blot was used to detect the inhibitory effect of G9a inhibitor on H3K9me2 in cardiomyocytes by different concentrations (0.1 µM, 0.5 μM, 1.0 μM and 2.0 μM). GAPDH and the total histone H3 were used as loading controls. (**C**) The relative mRNA expressions of mTOR in myocardial ischemia-reperfusion model *in vitro* were determined by qRT-PCR after adding 1.0 μM G9a inhibitor to Si group. (**D**) Western blot was used to detect the expression level of mTOR protein after adding 1.0 μM G9a inhibitor to Si group. GAPDH serves as a loading control. (**E**) Expression of mTOR protein relative to GAPDH data from 3 biological repeats is shown. (**F**) The relative mRNA expressions of Peak1-4 in Myocardial ischemia-reperfusion model *in vitro* were determined by qRT-PCR after adding 1.0 μM G9a inhibitor to Si group. Data shown are mean ± SD. *P < 0.05, **P < 0.01, ***P < 0.001, ****P < 0.0001. N=3 per group. Model, *in vitro* oxidative stress model; NC, negative control of RNAi; si, RNAi knockdown of Uhrf1; Uhrf1, Uhrf1 overexpression.

Uhrf1 can specifically recognize H3K9me2, which suppresses the expression of specific genes in target cells and tissues. Therefore, Uhrf1 can regulate the expression of specific genes by regulating H3K9me2. As previously reported, there are four peaks for H3K9me2 expression on the mTOR gene [4]. Thus, changes in the expression of peaks 1-4 when overexpressing and knocking-down Uhrf1 were investigated by qRT-PCR (Figure 6A). We found overexpression of Uhrf1 inhibits the four expression peaks of H3K9me2 on the mTOR gene and knocking down Uhrf1 promotes expression of these four peaks.

In order to further clarify the regulation of mTOR by H3K9me2, the H3K9me2 methyltransferase (G9a) inhibitor BIX01294 was added to the cardiomyocytes. The inhibitory effects of BIX01294 in various concentrations (0.1, 0.5, 1.0, and 2.0 μM) on cardiomyocyte H3K9me2 were evaluated, and the optimal inhibitory concentration was determined (Figure 6B). We observed that when the concentration of BIX01294 was 1.0 μM, the expression of H3K9me2 in cardiomyocytes was effectively inhibited. Changes in the mRNA and protein levels of mTOR were detected after adding G9a inhibitor (BIX01294) at 1.0 μM to the Si group (Figure 6C–6E). We observed that the expression of mTOR is increased in Uhrf1-knockdown cardiomyocytes after G9a inhibitor was added. The changes in the four expression peaks for H3K9me2 on mTOR after G9a inhibitor were further evaluated (Figure 6F). After G9a inhibitor was added, peak 1 and peak 2 decreased, while peak 3 and peak 4 did not change significantly. Thus, these results indicated that the regulation of mTOR expression by Uhrf1 was mediated by regulating H3K9me2.

### Effect of Uhrf1 on autophagy through mTOR in MIRI

**Figure 7:**
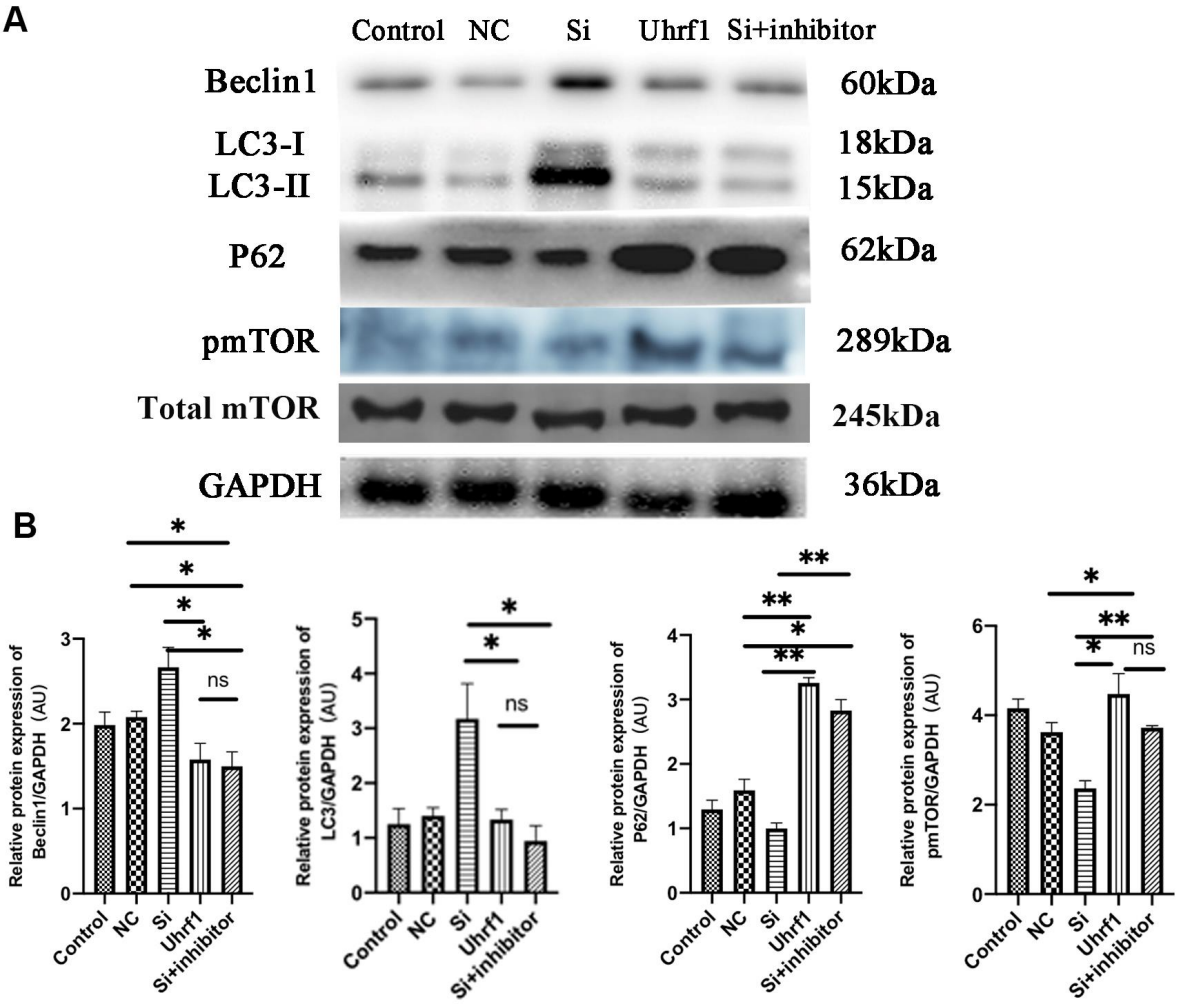
**The regulation of mTOR on autophagy in MIRI.** (**A**) Western blot was used to detect the expression level of Beclin1, LC-3, P62, total m-TOR and p-mTOR protein. GAPDH serves as a loading control. (**B**) Expression of Beclin1, LC-3, P62 and p-mTOR protein relative to GAPDH data from 3 biological repeats is shown. Data shown are mean ± SD. *P < 0.05, **P < 0.01, ***P < 0.001, ****P < 0.0001. N=3 per group. Model, *in vitro* oxidative stress model; NC, negative control of RNAi; si, RNAi knockdown of Uhrf1; Uhrf1, Uhrf1 overexpression.

To detect changes in autophagy after MIRI, the expression of autophagy-related proteins was evaluated (Figure 7A, 7B). After overexpression of Uhrf1, the ratio of LC3-II/LC3-I to Beclin-1 expression was significantly decreased, while the expression of P62 and p-mTOR was markedly increased. This indicated that Uhrf1 promotes the expression of mTOR during MIRI, thereby inhibits the occurrence of autophagy.

## DISCUSSION

According to the world health organization and the United Nations [5], ischemic heart disease, including acute myocardial infarction, is the major death reason worldwide. Therefore, finding endogenous strategies to reduce myocardial apoptosis and promote myocardial cell activity will offer new insights for the treatment of MIRI and improve overall survival rates. MIRI is a multiple step process, and epigenetic research provides a more comprehensive framework for understanding its pathology and biology [6]. Epigenetics refers to the inheritance of genetic information that is not based on DNA sequence but can be passed on to the next generation through DNA and histone modification and miRNA-dependent mechanisms [7]. There are a few kinds of post-translational modifications containing acetylation, methylation, phosphorylation, small ubiquitin-like modification, ubiquitination, carbonylation, glycosylation, and DNA methylation [8, 9]. Epigenetic modification factors are abnormally expressed during I/R-induced organ damage and may become potential biomarkers or therapeutic targets for I/R-injury-related diseases diagnosis [10]. In view of the significant role of epigenetic modification in the development and progression of cardiovascular and cerebrovascular diseases, we must reach a deeper understanding of the epigenetic events that promote or retard MIRI [11].

Uhrf1 is an epigenetic modifier with multiple fields and functions and is overexpressed in many cancers [12]. It leads to promoted cell proliferation and inhibited apoptosis in many tumor cells [13, 14]. However, the function of Uhrf1 in cardiomyocytes remains unclear. In this research, the effects of Uhrf1 on MIRI were explored *in vitro* and *in vivo*.

In the *in vivo* experiments, the expression of Uhrf1 in the ischemic and non-ischemic regions of the mouse heart was measured at 2, 6, and 12 hours after MIRI. Our results revealed that the level of Uhrf1 mRNA in the non-ischemic region of the mouse heart was significantly lower than that in the ischemic region. The increase of Uhrf1 in the ischemic area of the heart during MIRI is a manifestation of stress protection *in vivo*, which is short-term and limited. Thus, it is very important for clinical treatment to identify the mechanism of endogenous repair of MIRI.

In the *in vitro* experiments, Uhrf1 was demonstrated to inhibit cardiomyocyte apoptosis and increase cardiomyocyte viability when they are injured by H_2_O_2_ oxidative stress.

Through the detection of apoptotic factors, we found that after overexpression of Uhrf1, both Bax and caspase-3 exhibited lower expression levels and the expression level of Bcl-2 was higher. It was found that overexpression of Uhrf1 increased the number of cardiomyocytes in the proliferative cycle. Moreover, the expression of Ki67 at both the RNA and protein level decreased after Uhrf1 knockdown.

CCK8 was used to investigate cardiomyocyte activity. When Uhrf1 was overexpressed, the viability of the cardiomyocytes increased significantly, but when Uhrf1 was knocked down, the viability of cardiomyocytes did not decrease, suggesting that Uhrf1 can improve the viability of cardiomyocytes, and there are other compensatory mechanisms other than Uhrf1 that maintain the viability of cardiomyocytes when they are damaged.

We also observed that the number of beats per minute of the overexpressed Uhrf1 cardiomyocytes was higher than that of the reference group, while the beating rate of the knockdown Uhrf1 cardiomyocytes was slower. All of these results confirmed that Uhrf1 increases the viability of cardiomyocytes and the number of cardiomyocytes in the proliferation cycle. *In vitro* detection of the apoptosis and proliferation of cardiomyocytes further confirmed the protective effect of Uhrf1 in MIRI.

The Uhrf1 is able to activate the Akt-mTOR signaling pathway in iNKTs cells and increase mTOR expression [15]. Uhrf1 and mTOR also have an expression correlation during liver regeneration [16]. Furthermore, Wang et al*.* confirmed that Torin2 in hepatocellular carcinoma inhibits mTOR and also down-regulates Uhrf1 expression, which further illustrates the correlation between Uhrf1 and mTOR expression [17].

Similarly, our study confirms that Uhrf1 is related to mTOR during MIRI. Overexpression of Uhrf1 increases the expression of RAN and mTOR protein. Interestingly, the knockdown of Uhrf1 in MIRI only reduces the mTOR mRNA level, while the protein level did not decrease significantly. However, Uhrf1 mediated mTOR regulation has not been confirmed. We suspected that the regulation of mTOR mediated by Uhrf1 is related to the characteristics of the epigenetic factor of Uhrf1. Uhrf1 includes a SET-RING correlation (SRA) domain, which specifically identifies the hemi-methylated CpG, tandem Tudor domain (TTD), and plant homeodomain (PHD) combining the H3 tail with H3K9Me2/3. Based on the unique Uhrf1 structure, this chromatin modifier has been reported to be involved in DNA methylation and heterochromatin formation leading to gene silencing [18, 19]. H3K9me is a marker of gene silencing, and the TTD domain of Uhrf1 can specifically recognize and bind H3K9me2/3. Uhrf1 can recognize epigenetic markers through its special domain and combine with the corresponding catalytic enzyme to ensure stable inheritance. Recently, it has been reported that Uhrf1 can recognize hemi-methyl DNA and maintain H3K9me3-modified DNMT1 to maintain DNA methylation through the PBR domain [20]. Uhrf1 has a higher specific recognition and binding capacity for H3K9me2 in the human genome than H3K9me1 and H3K9me3 [21]. In addition, H3K9 dimethyltransferase G9a and Uhrf1 have a negative regulatory effect [22]. Therefore, Uhrf1 can inhibit or activate the expression of certain specific genes by regulating the expression of H3K9me2.

To study whether the regulatory effect of Uhrf1 on mTOR is related to histone H3K9me2, four highly enriched H3K9me2 fragments (peaks 1-4) in the mTOR gene were analyzed. We found that these fragments in Uhrf1-knockdown cardiomyocytes in MIRI significantly were increased. However, overexpression of Uhrf1 in the cardiomyocytes decreased the expression of these enriched fragments. Because histone H3K9me2 is a marker of epigenetic inhibitory expression, mTOR gene expression is suppressed when H3K9me2 highly-enriched fragments are highly expressed. Uhrf1 overexpression in MIRI leads to impaired expression of the epigenetic factor H3K9me2 on the mTOR gene and increased expression of mTOR. However, when Uhrf1 is knocked down, the expression of H3K9me2 on the mTOR gene is increased, which reduces the expression of mTOR.

In the current study, the H3K9 dimethylation inhibitor BIX01294 was added to Uhrf1-knockdown cardiomyocytes damaged by MIRI to inhibit histone H3K9 dimethylation. H3K9me2 inhibition in cardiomyocytes was found to be most obvious at a concentration of 1.0 μM. Furthermore, a significant increase in mTOR expression was detected after adding 1.0 μM of G9a inhibitor to the Uhrf1-knockdown group. In addition, the expressions of peak 1 and peak 2 also decreased, and peak 3 and peak 4 did not change significantly. Thus, we confirmed that the regulatory mechanism of Uhrf1 on mTOR involves the regulation of H3K9me2 on the mTOR gene.

As a family of phosphoinositide 3-kinase (PI3K) related kinases, mTOR forms two different complexes, which are called mTORC1 and mTORC2, by binding with a considerable number of chaperones [23, 24]. mTOR is essential for a variety of cellular processes, including cell growth, proliferation, survival, protein synthesis, autophagy, and metabolism. In the cardiovascular system, the mTOR signaling pathway integrates intracellular and extracellular signals as a central regulator of physiological and pathological processes [25]. There are two main mTOR-related signaling pathways, namely the AMPK-mTOR pathway and the PI3K-Akt-mTOR pathway [26]. Recent studies indicated that repair regulation occurs through these mTOR-related pathways. Vascular smooth muscle cells activate the PI3K/Akt pathway and attenuate myocardial I/R-induced apoptosis and autophagy by secreting bFGF [27]. Melatonin attenuates myocardial I/R injury by inhibiting autophagy via the AMPK/mTOR signaling pathway [28]. Thus, these studies provide strategies to reduce myocardial I/R injury by regulating mTOR-related signaling pathways. mTOR is an important factor in regulating autophagy and has been shown to regulate autophagy through different signaling pathways, thereby protecting and repairing MIRI. The current research confirmed that the regulation of mTOR by Uhrf1 affects the occurrence of autophagy during MIRI. Overexpression of Uhrf1 decreases the expression of LC3 and Beclin-1 and increases the expression of P62 and p-mTOR. Knockdown of Uhrf1 increased autophagy in MIRI, and this was inhibited when G9a inhibitor was added to the Uhrf1-knockdown group, indicating that Uhrf1 further regulates autophagy through the regulation of mTOR by H3K9me2.

Autophagy is a general term for the lysosomal-dependent degradation of material components in cells and it is unique to eukaryotic cells [29, 30]. Autophagy plays a double-edged role in MIRI, and it may have a negative effect during the reperfusion phase. Although reperfusion provides an energy substrate for the heart, it is reported that tissue damage is paradoxically intensified by increasing the production of cytosolic Ca^2+^ and ROSs. Despite autophagy being a compensation mechanism to counteract nutritional deficiencies in the ischemic phase, the continued increase in AMPK activation during reperfusion can lead to chronic autophagic activity and cell death [31]. Wei et al*.* confirmed that autophagy in the ischemic phase is beneficial, while excessive autophagy in the reperfusion phase can induce the gradual consumption of cellular components, leading to autophagic cell death [32]. In this study, the protective effect of Uhrf1 in MIRI was demonstrated to occur by reducing the autophagy of cardiomyocytes.

As mentioned above, we demonstrated the repair effect of Uhrf1 overexpression in MIRI. Uhrf1 regulates mTOR expression by regulating H3K9me2 modification, and the regulation of mTOR by Uhrf1 during MIRI affects the occurrence of autophagy. In conclusion, our current study indicates the potential role of the Uhrf1/mTOR / autophagy axis in MIRI protection.

## MATERIALS AND METHODS

### Animals

In this study, C57BL/6J mice were purchased from Jackson laboratory. The mice were placed under the standard conditions of animals with 21 ± 1° C temperature and 55%-60% humidity, and they ate and drank at will. In the experiment, C57BL/6J male mice (25-30 g) were selected for *in vivo* experiment and newborn mice (1-3 days old) for *in vitro* experiment. Experimental procedures were carried out in accordance with the animal care and use system approved by the Committee of Harbin Medical University.

### Myocardial ischemia/reperfusion model preparation

According to the "Guidelines for the Care and Use of Laboratory Animals" published by the National Institutes of Health (NIH Publication No. 85-23, revised 1996), animal experiments were performed. Nine male C57BL/6J mice aged 2-3 months (25-30 g) were divided into three groups: 2, 6, and 12 hours. A 100% concentrated storage solution was prepared by mixture of 10 g avertin and 10 mL tert-amyl alcohol. When used, it was diluted to 1.2% with sterilized distilled water, and the dosage was 0.7 mL/20 g for intraperitoneal injection of anesthetized mice, which was also used as an analgesic during the experiment. The left anterior descending coronary artery was ligated with a loose knot (6-0 wires). After 30 minutes of ischemia, the loose knots were released. After I/R operation, air is discharged from the chest and the surgical wound is sutured. The hearts of the mice were taken at 2, 6, and 12 hours (according to group) after reperfusion, and the ischemic and non-ischemic areas were separated.

### Cardiomyocyte culture

Newborn mice were taken, and a primary culture of neonatal mouse ventricular myocytes was prepared by enzymatic hydrolysis of cardiac tissue. Briefly, the hearts were enzymatically digested in 0.25% trypsin and centrifuged at 1500 r/min for 5 minutes. The cardiomyocytes were cultured at 37° C for 90 minutes for cardiomyocyte purification, and the resultant cardiomyocytes were cultured at a concentration of 1 × 10^5^/mL in DMEM supplemented with 10% fetal bovine serum (FBS) and 100 U/mL of penicillin and 100 mg/mL of streptomycin for 48 hours in an incubator (37° C, 5% CO_2_).

### Experimental grouping

The isolated cardiomyocytes were sorted into cardiomyocyte, model, blank plasmid, knock-down Uhrf1, over-expressing Uhrf1 and Si with H3K9me2 methyltransferase (G9a) inhibitor (BIX01294: Cat.No. HY-10587, MedChemExpress, China) groups. Reagent addition for each group is given in Table 2.

**Table 2:** Reagents used for each *in vitro* analysis group.

	**Control group**	**Model group**	**NC group**	**Si group**	**Uhrf1 group**	**Si+inhibitor group**
Primary cardiomyocytes	+	+	+	+	+	+
H_2_O_2_	/	+	+	+	+	+
Non-specific control plasmid	/	/	+	/	/	/
si-m-Uhrf1 plasmid	/	/	/	+	/	+
Uhrf1 plasmid	/	/	/	/	+	/
G9a inhibitor(BIX01294)	/	/	/	/	/	+

### Plasmid transfection

For amplification and extraction of Uhrf1 DNA plasmid pRP[Exp]-EGFP/Puro-CAG>mUhrf1 [NM_010931.3]* (Vector Builder), 2000 ng of the Uhrf1 plasmid was mixed with 15 μL of X-treme GENE siRNA Transfection Reagent (Roche, Basel, Switzerland) and dissolved in opti-MEM medium. The mixed liquid was added to the cardiomyocytes at 100 μL/mL culture solution. The siRNA sequence of Uhrf1 was labeled CCTTGCAGACCATTCTCAA, and 7 μL of this siRNA plasmid and blank NC plasmid (purchased from RIBBIO) were mixed with 10 μL of x-treme and dissolved in opti-MEM. The mixed liquid was added to the cardiomyocytes at 100 μL/mL culture solution. The cells were incubated for 24 hours.

### Induction of *in vitro* oxidative stress model

After 24 hours of plasmid transfection, 3% H_2_O_2_ (1 mol/L) was diluted 100-fold to a final concentration of 10 nmol/μL and passed through a 0.22 μm filter. This was then added 100 nmol H_2_O_2_ per 1 mL of cardiomyocyte culture solution. Oxidative stress was induced by hydrogen peroxide in serum-free medium for 24 hours, and then exposed to normoxic conditions and DMEM containing glucose for the next 24 hours.

### Quantitative real-time PCR

Total RNA was isolated using TRIzol reagent (Invitrogen, Carlsbad, CA, USA), and cDNA samples were obtained by reverse transcription using TransScript All-in-One reagent (TransGen Biotech, China). The expression levels of specific genes and GAPDH was determined by quantitative real-time PCR (qRT-PCR) in a CFX96 real-time PCR system. The results of qRT-PCR were normalized to GAPDH expression. Use method 2^-∆∆Ct^ to calculate the relative quantitative expression. All reactions were repeated three times. The sequences of primers are listed in the Table 3.

**Table 3:** Primer sequences used in this study.

**Gene**	**Primer sequence**
Bax	F: 5’-TCATGAAGACAGGGGCCTTT -3’ R: 5’-GTCCACGTCAGCAATCATCC-3’
Bcl-2	F: 5’- CTTCAGGGATGGGGTGAACT-3’ R: 5’-CAGCCTCCGTTATCCTGGAT-3’
Caspase-3	F: 5’-CTCGCTCTGGTACGGATGTG -3’ R: 5’-TCCCATAAATGACCCCTTCATCA-3’
Ki67	F: 5’-CGCAGGAAGACTCGCAGTTT-3’ R: 5’-CTGAATCTGCTAATGTCGCCAA-3’
mTOR	F: 5’-CTGTAATTACATCCTCGACTG-3’ R: 5’-CGTGTCGTGGTTAGTCG-3’
Uhrf1	F: 5’-AGCAAGCAAAGTCCACAGG -3’ R: 5’-TTAGCACATCCCACAGC-3’
Peak1	F: 5’- CTGAGGAGACGGGATTCAGG-3’ R: 5’- GGAACCCAGGGCTGAACTAC-3’
Peak2	F: 5’- AAAGAGTGGTTCGTGGCGTC -3’ R: 5’- ACCCC TAGAGTGAGGTGTGT-3’.
Peak3	F: 5’- AGATTGGTCGTCAGTAGGCAC -3’ R: 5’- TCTGGCACTGCAGTTTGGTT-3’
Peak4	F: 5’- TTGCACCCTCACCCCTTTTC -3’ R: 5’- GCTAACCGGTCATTCCCTCA-3’
GAPDH	F: 5’-CGCTCTCTGCTCCTCCTGTTC-3’ R: 5’-ATCCGTTGACTCCGACCTTCAC-3’

F: forward; R: reverse

### Protein extraction and western blot

Cardiomyocytes were lysed in RIPA buffer (150 mM NaCl, 10% glycerol, 50 mM Tris/HCl, and 1% NP-40, pH 8.0) containing phosphatase and protease inhibitors for 30 minutes. Cardiomyocyte fragments were removed by centrifugation at 12,000 rpm for 20 minutes at 4° C. Protein concentrations were determined using a Thermo Pierce BCA Protein Assay Kit. A 50 μg protein sample was boiled in the loading buffer, denatured, and fractionated by SDS-PAGE. Then, the gel containing the protein was placed on a PVDF membrane in a membrane transport tank. After sealing with 5% skimmed milk, the PVDF membrane was incubated overnight with antibodies of Uhrf1, Bax, caspase-3, Bcl-2, Ki67 and mTOR from Bioss (Beijing, China), Beclin-1, P62, LC3 from Proteintech (Wuhan, China), p-mTOR and H3K9me2 from Immunoway (Newark, USA) and GAPDH from Goodhere Biotechnology (Hangzhou, China). On the second day, the second-order fluorescent antibody was incubated for 2 hours. The immune response band was detected by Protein Simple system.

### Immunocytochemistry

First, 1 mL of 4% paraformaldehyde was fixed for 15 minutes, and 0.3% Triton at 1 mL/well membrane was added. After 10 minutes, the capsules were washed with goat serum for 15-20 minutes. Meanwhile, 50 μL of primary antibody was diluted with 1% BSA and placed in a wet box at 4° C overnight. Then, the wet box was removed and the small slides were placed back into a 12 well plate and each well was rinsed with phosphate-buffered saline (PBS) three times. Then, in a dark room, 50 μL of the secondary antibody diluted with 1% BSA was added and the plate was preheated at 37° C. The incubation of the secondary antibody was completed after 1 hours. The nuclei were stained with DAPI for approximately 5 minutes and glycerin seals were applied. Under the microscope, three different fields of vision were used to randomly select each slide for scoring. The intensity and positive proportion were semi-quantitatively scored. The intensity score was used to calculate protein expression level.

### CCK8 cell viability test

The cardiomyocytes were inoculated into 96 well culture plate, with 1 × 10^4^ cells per well. The plates were pre-cultured in an incubator for 24 hours (37° C, 5% CO_2_). Cells in each well were treated with 10 μL of CCK8 reagent (Dojindo) with care taken not to generate bubbles in the pore as they affect the OD value. The plates were incubated in an incubator for 2 hours. The absorbance at 450 nm was measured.

### Statistical analyses

Statistical calculation and analysis of counting test results and data were performed and evaluated by ANOVA or Student's t-test for two or multiple group comparisons by using GraphPad Prism 8. The difference in the detection indexes was considered statistically significant when *P*<0.05. The data in the experiment were expressed as mean ± SD.

## References

[1] Yang CF. Clinical manifestations and basic mechanisms of myocardial ischemia/reperfusion injury. Ci Ji Yi Xue Za Zhi. 2018; 30:209–15. 10.4103/tcmj.tcmj_33_18 30305783 PMC6172894

[2] Bellanti F. Hypoxia-inducible factor-1 in myocardial ischaemia/reperfusion injury. Acta Physiol (Oxf). 2017; 221:93–94. 10.1111/apha.12903 28581154

[3] Zhang ZX, Li H, He JS, Chu HJ, Zhang XT, Yin L. Remote ischemic postconditioning alleviates myocardial ischemia/reperfusion injury by up-regulating ALDH2. Eur Rev Med Pharmacol Sci. 2018; 22:6475–84. 10.26355/eurrev_201810_16061 30338817

[4] Gidlöf O, Johnstone AL, Bader K, Khomtchouk BB, O’Reilly JJ, Celik S, Van Booven DJ, Wahlestedt C, Metzler B, Erlinge D. Ischemic preconditioning confers epigenetic repression of mtor and induction of autophagy through G9a-dependent H3K9 dimethylation. J Am Heart Assoc. 2016; 5:e004076. 10.1161/JAHA.116.004076 28007739 PMC5210409

[5] Xie Y, Ji R, Han M. Eriodictyol protects H9c2 cardiomyocytes against the injury induced by hypoxia/reoxygenation by improving the dysfunction of mitochondria. Exp Ther Med. 2019; 17:551–57. 10.3892/etm.2018.6918 30651835 PMC6307419

[6] Kim H, Wang X, Jin P. Developing DNA methylation-based diagnostic biomarkers. J Genet Genomics. 2018; 45:87–97. 10.1016/j.jgg.2018.02.003 29496486 PMC5857251

[7] Khan O, La Thangue NB. HDAC inhibitors in cancer biology: emerging mechanisms and clinical applications. Immunol Cell Biol. 2012; 90:85–94. 10.1038/icb.2011.100 22124371

[8] Nightingale KP, O’Neill LP, Turner BM. Histone modifications: signalling receptors and potential elements of a heritable epigenetic code. Curr Opin Genet Dev. 2006; 16:125–36. 10.1016/j.gde.2006.02.015 16503131

[9] Bolden JE, Peart MJ, Johnstone RW. Anticancer activities of histone deacetylase inhibitors. Nat Rev Drug Discov. 2006; 5:769–84. 10.1038/nrd2133 16955068

[10] Tang J, Zhuang S. Histone acetylation and DNA methylation in ischemia/reperfusion injury. Clin Sci (Lond). 2019; 133:597–609. 10.1042/CS20180465 30804072 PMC7470454

[11] van der Harst P, de Windt LJ, Chambers JC. Translational perspective on epigenetics in cardiovascular disease. J Am Coll Cardiol. 2017; 70:590–606. 10.1016/j.jacc.2017.05.067 28750703 PMC5543329

[12] Ashraf W, Ibrahim A, Alhosin M, Zaayter L, Ouararhni K, Papin C, Ahmad T, Hamiche A, Mély Y, Bronner C, Mousli M. The epigenetic integrator UHRF1: on the road to become a universal biomarker for cancer. Oncotarget. 2017; 8:51946–62. 10.18632/oncotarget.17393 28881702 PMC5584303

[13] Hu Q, Qin Y, Ji S, Xu W, Liu W, Sun Q, Zhang Z, Liu M, Ni Q, Yu X, Xu X. UHRF1 promotes aerobic glycolysis and proliferation via suppression of SIRT4 in pancreatic cancer. Cancer Lett. 2019; 452:226–36. 10.1016/j.canlet.2019.03.024 30905812

[14] Jiao D, Huan Y, Zheng J, Wei M, Zheng G, Han D, Wu J, Xi W, Wei F, Yang AG, Qin W, Wang H, Wen W. UHRF1 promotes renal cell carcinoma progression through epigenetic regulation of TXNIP. Oncogene. 2019; 38:5686–99. 10.1038/s41388-019-0822-6 31043707

[15] Cui Y, Chen X, Zhang J, Sun X, Liu H, Bai L, Xu C, Liu X. Uhrf1 controls iNKT cell survival and differentiation through the Akt-mTOR axis. Cell Rep. 2016; 15:256–63. 10.1016/j.celrep.2016.03.016 27050515

[16] Bracht T, Hagemann S, Loscha M, Megger DA, Padden J, Eisenacher M, Kuhlmann K, Meyer HE, Baba HA, Sitek B. Proteome analysis of a hepatocyte-specific BIRC5 (survivin)-knockout mouse model during liver regeneration. J Proteome Res. 2014; 13:2771–82. 10.1021/pr401188r 24818710

[17] Wang C, Wang X, Su Z, Fei H, Liu X, Pan Q. The novel mTOR inhibitor torin-2 induces autophagy and downregulates the expression of UHRF1 to suppress hepatocarcinoma cell growth. Oncol Rep. 2015; 34:1708–16. 10.3892/or.2015.4146 26239364

[18] Hashimoto H, Horton JR, Zhang X, Cheng X. UHRF1, a modular multi-domain protein, regulates replication-coupled crosstalk between DNA methylation and histone modifications. Epigenetics. 2009; 4:8–14. 10.4161/epi.4.1.7370 19077538 PMC2661099

[19] Bronner C, Krifa M, Mousli M. Increasing role of UHRF1 in the reading and inheritance of the epigenetic code as well as in tumorogenesis. Biochem Pharmacol. 2013; 86:1643–49. 10.1016/j.bcp.2013.10.002 24134914

[20] Gao L, Tan XF, Zhang S, Wu T, Zhang ZM, Ai HW, Song J. An intramolecular interaction of UHRF1 reveals dual control for its histone association. Structure. 2018; 26:304–11.e3. 10.1016/j.str.2017.12.016 29395786 PMC5803408

[21] Abhishek S, Nivya MA, Nakarakanti NK, Deeksha W, Khosla S, Rajakumara E. Biochemical and dynamic basis for combinatorial recognition of H3R2K9me2 by dual domains of UHRF1. Biochimie. 2018; 149:105–14. 10.1016/j.biochi.2018.04.010 29656054

[22] Kim KB, Son HJ, Choi S, Hahm JY, Jung H, Baek HJ, Kook H, Hahn Y, Kook H, Seo SB. H3K9 methyltransferase G9a negatively regulates UHRF1 transcription during leukemia cell differentiation. Nucleic Acids Res. 2015; 43:3509–23. 10.1093/nar/gkv183 25765655 PMC4402520

[23] Huang WQ, Wen JL, Lin RQ, Wei P, Huang F. Effects of mTOR/NF-κB signaling pathway and high thoracic epidural anesthesia on myocardial ischemia-reperfusion injury via autophagy in rats. J Cell Physiol. 2018; 233:6669–78. 10.1002/jcp.26320 29206300

[24] Okamoto T, Ozawa Y, Kamoshita M, Osada H, Toda E, Kurihara T, Nagai N, Umezawa K, Tsubota K. The neuroprotective effect of rapamycin as a modulator of the mTOR-NF-κB axis during retinal inflammation. PLoS One. 2016; 11:e0146517. 10.1371/journal.pone.0146517 26771918 PMC4714903

[25] Samidurai A, Kukreja RC, Das A. Emerging role of mTOR signaling-related miRNAs in cardiovascular diseases. Oxid Med Cell Longev. 2018; 2018:6141902. 10.1155/2018/6141902 30305865 PMC6165581

[26] Lin XL, Xiao WJ, Xiao LL, Liu MH. Molecular mechanisms of autophagy in cardiac ischemia/reperfusion injury (review). Mol Med Rep. 2018; 18:675–83. 10.3892/mmr.2018.9028 29845269

[27] Ye G, Fu Q, Jiang L, Li Z. Vascular smooth muscle cells activate PI3K/Akt pathway to attenuate myocardial ischemia/reperfusion-induced apoptosis and autophagy by secreting bFGF. Biomed Pharmacother. 2018; 107:1779–85. 10.1016/j.biopha.2018.05.113 30257397

[28] Chen WR, Liu HB, Chen YD, Sha Y, Ma Q, Zhu PJ, Mu Y. Melatonin attenuates myocardial ischemia/reperfusion injury by inhibiting autophagy via an AMPK/mTOR signaling pathway. Cell Physiol Biochem. 2018; 47:2067–76. 10.1159/000491474 29975938

[29] Saha S, Panigrahi DP, Patil S, Bhutia SK. Autophagy in health and disease: a comprehensive review. Biomed Pharmacother. 2018; 104:485–95. 10.1016/j.biopha.2018.05.007 29800913

[30] Xie W, Zhou J. Aberrant regulation of autophagy in mammalian diseases. Biol Lett. 2018; 14:20170540. 10.1098/rsbl.2017.0540 29321247 PMC5803588

[31] Daniels LJ, Varma U, Annandale M, Chan E, Mellor KM, Delbridge LM. Myocardial energy stress, autophagy induction, and cardiomyocyte functional responses. Antioxid Redox Signal. 2019; 31:472–86. 10.1089/ars.2018.7650 30417655

[32] Wei K, Wang P, Miao CY. A double-edged sword with therapeutic potential: an updated role of autophagy in ischemic cerebral injury. CNS Neurosci Ther. 2012; 18:879–86. 10.1111/cns.12005 22998350 PMC6493521

